# TarBase-v9.0 extends experimentally supported miRNA–gene interactions to cell-types and virally encoded miRNAs

**DOI:** 10.1093/nar/gkad1071

**Published:** 2023-11-20

**Authors:** Giorgos Skoufos, Panos Kakoulidis, Spyros Tastsoglou, Elissavet Zacharopoulou, Vasiliki Kotsira, Marios Miliotis, Galatea Mavromati, Dimitris Grigoriadis, Maria Zioga, Angeliki Velli, Ioanna Koutou, Dimitra Karagkouni, Steve Stavropoulos, Filippos S Kardaras, Anna Lifousi, Eustathia Vavalou, Armen Ovsepian, Anargyros Skoulakis, Sotiris K Tasoulis, Spiros V Georgakopoulos, Vassilis P Plagianakos, Artemis G Hatzigeorgiou

**Affiliations:** DIANA-Lab, Dept. of Computer Science and Biomedical Informatics, University of Thessaly, Lamia, Greece; Hellenic Pasteur Institute, Athens11521, Greece; Dept. of Informatics and Telecommunications, National and Kapodistrian Univ. of Athens, Athens, Greece; Biomedical Research Foundation of the Academy of Athens, 11527Athens, Greece; DIANA-Lab, Dept. of Computer Science and Biomedical Informatics, University of Thessaly, Lamia, Greece; Hellenic Pasteur Institute, Athens11521, Greece; DIANA-Lab, Dept. of Computer Science and Biomedical Informatics, University of Thessaly, Lamia, Greece; Hellenic Pasteur Institute, Athens11521, Greece; DIANA-Lab, Dept. of Computer Science and Biomedical Informatics, University of Thessaly, Lamia, Greece; Hellenic Pasteur Institute, Athens11521, Greece; DIANA-Lab, Dept. of Computer Science and Biomedical Informatics, University of Thessaly, Lamia, Greece; Hellenic Pasteur Institute, Athens11521, Greece; DIANA-Lab, Dept. of Computer Science and Biomedical Informatics, University of Thessaly, Lamia, Greece; DIANA-Lab, Dept. of Computer Science and Biomedical Informatics, University of Thessaly, Lamia, Greece; DIANA-Lab, Dept. of Computer Science and Biomedical Informatics, University of Thessaly, Lamia, Greece; DIANA-Lab, Dept. of Computer Science and Biomedical Informatics, University of Thessaly, Lamia, Greece; DIANA-Lab, Dept. of Computer Science and Biomedical Informatics, University of Thessaly, Lamia, Greece; DIANA-Lab, Dept. of Computer Science and Biomedical Informatics, University of Thessaly, Lamia, Greece; Hellenic Pasteur Institute, Athens11521, Greece; Department of Computer Science and Biomedical Informatics, University of Thessaly, Lamia, Greece; DIANA-Lab, Dept. of Computer Science and Biomedical Informatics, University of Thessaly, Lamia, Greece; Hellenic Pasteur Institute, Athens11521, Greece; Technical University of Denmark – Department of Health Technology, Copenhagen, Denmark; Department of Biology, National and Kapodistrian University of Athens, 15784Athens, Greece; DIANA-Lab, Dept. of Computer Science and Biomedical Informatics, University of Thessaly, Lamia, Greece; Hellenic Pasteur Institute, Athens11521, Greece; DIANA-Lab, Dept. of Computer Science and Biomedical Informatics, University of Thessaly, Lamia, Greece; Hellenic Pasteur Institute, Athens11521, Greece; Department of Computer Science and Biomedical Informatics, University of Thessaly, Lamia, Greece; Department of Mathematics, University of Thessaly, Greece; Department of Computer Science and Biomedical Informatics, University of Thessaly, Lamia, Greece; DIANA-Lab, Dept. of Computer Science and Biomedical Informatics, University of Thessaly, Lamia, Greece; Hellenic Pasteur Institute, Athens11521, Greece

## Abstract

TarBase is a reference database dedicated to produce, curate and deliver high quality experimentally-supported microRNA (miRNA) targets on protein-coding transcripts. In its latest version (v9.0, https://dianalab.e-ce.uth.gr/tarbasev9), it pushes the envelope by introducing virally-encoded miRNAs, interactions leading to target-directed miRNA degradation (TDMD) events and the largest collection of miRNA–gene interactions to date in a plethora of experimental settings, tissues and cell-types. It catalogues ∼6 million entries, comprising ∼2 million unique miRNA–gene pairs, supported by 37 experimental (high- and low-yield) protocols in 172 tissues and cell-types. Interactions are annotated with rich metadata including information on genes/transcripts, miRNAs, samples, experimental contexts and publications, while millions of miRNA-binding locations are also provided at cell-type resolution. A completely re-designed interface with state-of-the-art web technologies, incorporates more features, and allows flexible and ingenious use. The new interface provides the capability to design sophisticated queries with numerous filtering criteria including cell lines, experimental conditions, cell types, experimental methods, species and/or tissues of interest. Additionally, a plethora of fine-tuning capacities have been integrated to the platform, offering the refinement of the returned interactions based on miRNA confidence and expression levels, while boundless local retrieval of the offered interactions and metadata is enabled.

## Graphical Abstract

**Graphical Abstract:**
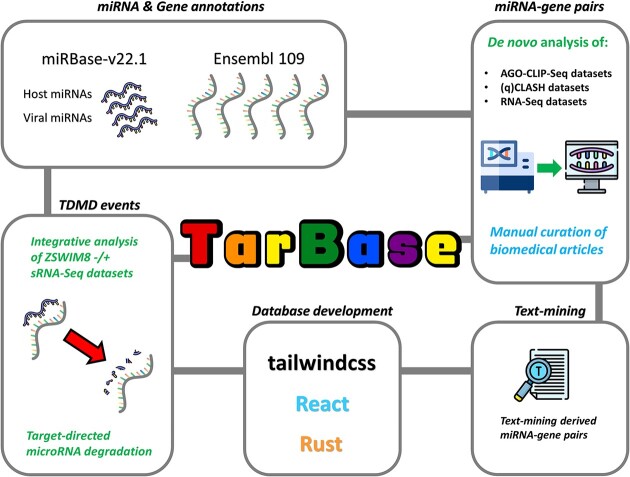

## Introduction

miRNAs play important roles in many aspects of molecular biology in both physiological and pathological conditions including cardiovascular and neural development, stem cell differentiation, metabolism, apoptosis, neurodegenerative diseases and tumors (1–5). A large body of evidence from recently published studies suggest that miRNAs also have alternative functions; in some cases, miRNAs have been identified to upregulate protein expression (6), activate transcription by direct interaction with DNA (7), target non-coding RNAs in the cell nucleus (8) and be highly enriched in the extracellular space (9). Their presence in the extracellular space (e.g. blood plasma), inside exosome-like extracellular micro-vesicles, has been extensively studied under the contexts of cell–cell signalling and biomarker potential (10,11). Additionally, evidence suggests that miRNAs may also partake in paracrine signalling, binding Toll-like receptors (12,13).

Since their initial discovery, numerous experimental and computational methods have been developed aiming at the identification, annotation, *de novo* prediction and cataloguing of miRNA targets on coding and non-coding transcripts. AGO-CLIP-Seq protocols (14,15) (e.g. HITS-CLIP, PAR-CLIP) have been widely adopted, offering context-specific miRNA interactomes in a transcriptome-wide manner. Furthermore, CLEAR-CLIP (16), CLASH (17) and qCLASH (18) protocols blaze the trail towards direct observations of miRNA-RNA pairs by utilizing a ligation step that links miRNAs with their RNA targets resulting in chimeric RNA fragments prior to sequencing. Additional methods with focus in the identification of miRNA–gene pairs have been employed; such methods include miRNA transfection/knockout cells followed by Microarray, RNA-Seq, RPF-Seq and RIP-Seq experiments, RT-qPCR, Western blot and Luciferase Reporter Assays and others.

Except for the miRNA-induced degradation and translational suppression of their targets, another major emerging process termed target-directed miRNA degradation (TDMD), promotes the destruction of miRNAs instead (19,20). Mechanistically, TDMD events start by the extensive pairing of the miRNA 3′ region to the target RNA molecule; this results in the destabilization of the miRNA which in turn is recognized by the ZSWIM8 ubiquitin ligase. ZSWIM8 adds polyubiquitin chains to AGO, leading to its proteolysis and subsequently exposing the miRNA to degradation.

The systematic curation and indexing of the rapidly expanding volume of miRNA–gene interactions in different experimental settings and species is vital to basic and applied RNA research and is addressed by TarBase and other online repositories. miRTarBase (21) is a database devoted to miRNA–gene interactions. The database comprises >2 million miRNA–target pairs from low- and high-throughput methodologies through manual curation of the available literature and the analysis of CLIP-Seq datasets. ENCORI/StarBase (22) is an online platform focused on RNA-RNA and protein-RNA interactions. The database contains more than 1 million miRNA-mRNA interactions and additional interactions between other RNA species and proteins. MiRecords (23) is another database with similar content, albeit with infrequent updates (last updated in 2009) and significantly fewer interactions (i.e. ∼3000 entries).

**Figure 1.**
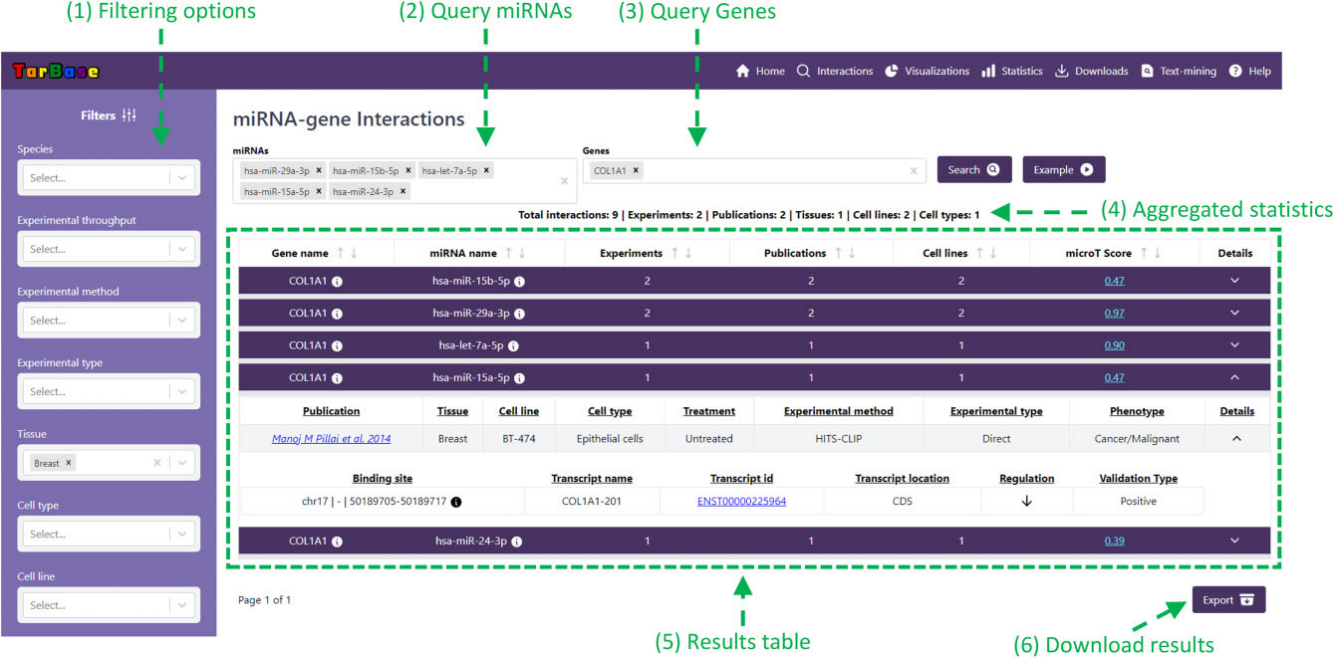
TarBase-v9.0 main user interface. (1) TarBase offers numerous filtering options (i.e. cell lines, experimental conditions, cell types etc.). Users may conduct queries with one or more miRNA(s) (2) and/or one or more genes (3). (4) Aggregated statistics of the returned interactions, (5) Results table and (6) an option to download the returned interactions.

We present TarBase-v9.0 (Figure 1), an extensive online repository of ∼6 million entries, 3-fold more than miRTarBase and 6-fold more than TarBase-v8.0, making it the largest collection of experimentally supported miRNA–gene interactions to date. It is the first TarBase version to employ microCLIP (24), an *avant-garde* CLIP-Seq analysis framework that combines deep learning classifiers under a super learning scheme for CLIP-Seq-guided detection of miRNA interactions. TarBase-v9.0 also incorporates interactions from direct miRNA–target chimeras produced by the *de novo* analysis of CLASH and qCLASH experiments, as well as miRNA–gene pairs derived from the analysis of miRNA-specific transfection/knockdown RNA-Seq experiments with a standardized data processing pipeline. Importantly, TarBase-v9.0 introduces (i) ∼34 000 interacting pairs between host mRNAs and virally-encoded miRNAs, (ii) interactions leading to target-directed miRNA degradation (TDMD) events and (iii) millions of exact miRNA-binding locations with cell-type resolution.

TarBase interface has been entirely revamped, introducing an array of additional features that enhance its versatility and usability. The newly introduced interface empowers users to design sophisticated queries, offering a multitude of filtering options such as cell lines, experimental conditions, cell types, experimental techniques, species, and tissues of interest. Furthermore, an abundance of fine-tuning capacities has been seamlessly integrated into the platform, enabling users to refine their search results based on miRNA confidence, expression levels, as well as microCLIP and DIANA-microT 2023 (25) scores. Additionally, the platform now facilitates unrestricted local retrieval of both the interactions and all associated metadata.

## Methods and results

### Database statistics and content

**Figure 2.**
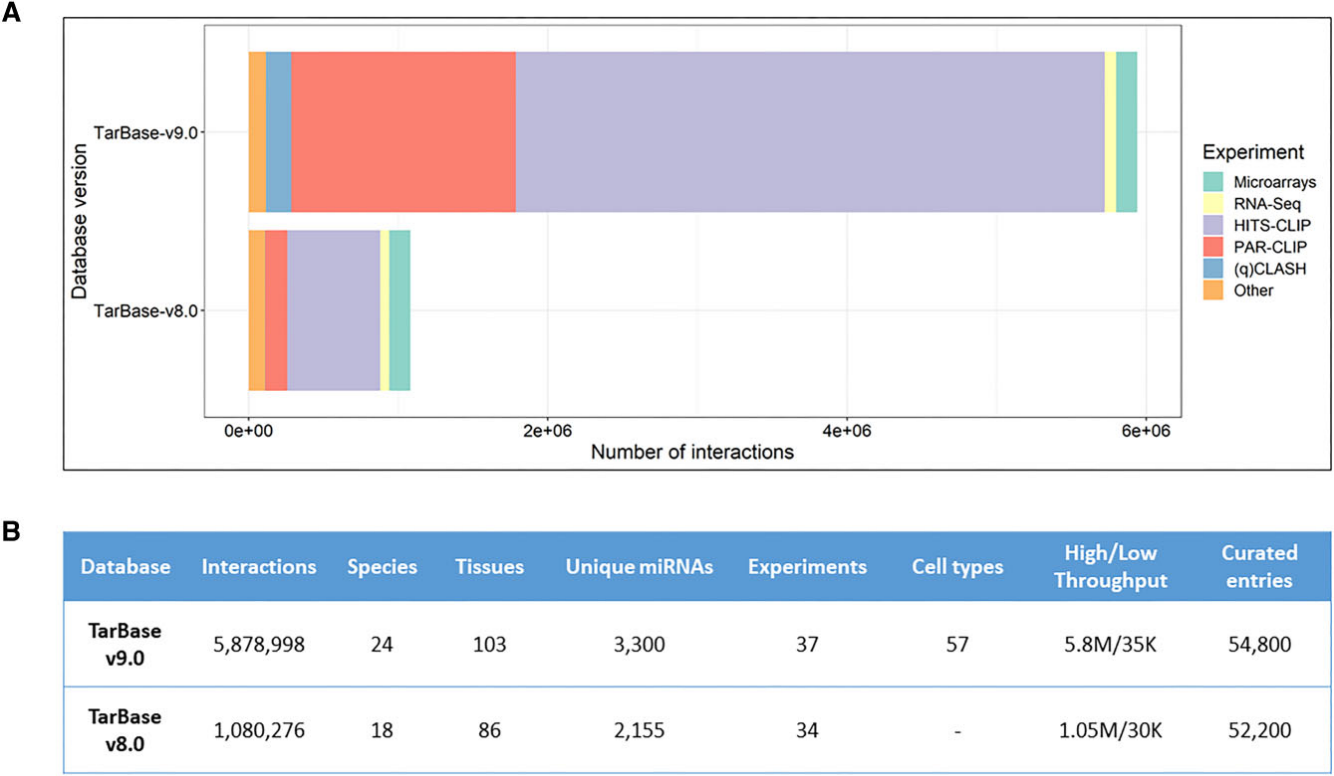
Comparison of TarBase-v9.0 versus TarBase-v8.0 in terms of (**A**) total interactions per experimental method and (**B**) other basic statistics of the databases.

*De novo* analysis of Next-Generation Sequencing (NGS) protocols including AGO-PAR-CLIP, CLASH and RNA-Seq samples and the manual curation of biomedical literature yielded a total of ∼6 million entries (Figure 2). These interactions comprise ∼2 million unique miRNA–gene pairs spanning over 103 tissues, 57 cell-types and >300 cell-lines. In total, TarBase features interactions derived from 22 low- and 15 high-throughput experimental techniques. Furthermore, TarBase-v9.0 incorporates >3300 miRNAs and >40 000 genes spreading over 24 species (e.g. *Homo sapiens, Mus musculus, Danio rerio, Drosophila melanogaster* etc.). Moreover, for every annotated miRNA–gene pair, the database integrates additional information including article metadata, details on genes and miRNAs (e.g. Ensembl (26) and miRBase (27) names and stable identifiers, the miRNA sequence, the targeted region genomic coordinates etc.), sample treatments, phenotypes, microT-CDS-2023 (25) predicted scores and more.

TarBase-v9.0 provides a ∼6-fold increase in total and a ∼3-fold increase in unique miRNA–gene interactions compared to TarBase-v8.0 (28). Additionally, it features >45 000 candidate Target-Directed miRNA Degradation events which to the best of our knowledge is the first ever biomedical database to host such information in a well curated collection. Finally, for the first time, boundless local retrieval of the entirety of TarBase interactions and metadata is offered to users.

### Database functionality and interface

TarBase-v9.0 interface has been redesigned from the ground up, bringing in numerous new features that improve its flexibility and user-friendliness. The development of the latest TarBase was conducted using state-of-the-art web technologies, enabling users to conduct queries with lightning-fast response times. Interactions can be retrieved by performing queries with miRNA or gene names/identifiers or both simultaneously. A plethora of filtering options to refine the returned miRNA–target pairs can be applied for species, experimental method, experimental type (i.e. direct or indirect protocols), cell line, tissue, cell type and gene region (i.e. 3′UTR or CDS). Additional fine-tuning can be employed with regard to miRNA expression, microT-CDS-2023 (25) prediction scores, miRNA confidence (miRBase-v22.1), binding site information and year of publication.

TarBase-v9.0 is also seamlessly interconnected with other biomedical databases. Since its sixth version, TarBase has been integrated in Ensembl (26). Interactions with exact binding sites can be viewed in the Ensembl genome browser; gene and miRNA information can be accessed through Ensembl and miRBase (27) respectively. TarBase is also included as an expert non-coding RNA resource in the RNACentral knowledgebase. Finally, information on publications can be directly retrieved through the interconnection with PubMed while miRNA–target interaction scores and predicted binding locations can also be reached by DIANA-microT-CDS 2023.

Furthermore, a separate page comprising miRNA–gene interactions (at miRNA precursor level) which have been automatically extracted via an auxiliary text mining pipeline with full-text capacity is incorporated to the interface. Basic statistics of the database (e.g. top experimental methods) are presented through bar-plots in a dedicated page. A visualization page to assess the combinatorial effect of multiple miRNAs in one or multiple genes is also provided; using this feature, users may conduct queries with two or more miRNAs and a visual representation of the interactions between the selected miRNAs and their respected targets will be rendered in the form of a network graph. For a gene to be included in the network graph, it must interact with at least two of the selected miRNAs. Finally, a help page with rich material (screenshots and text-based walkthroughs) is available to guide users and ensure a smooth experience for first-time visitors.

### Analysis of high-throughput datasets

**AGO-CLIP-Seq**. Raw AGO-CLIP-Seq libraries were quality checked and pre-processed using FastQC (www.bioinformatics.babraham.ac.uk/projects/fastqc/) and cutadapt (29). Subsequently, CLIP-Seq samples were aligned against the human and mouse reference genomes (i.e. GRCh38 and mm10) with GMAP/GSNAP (30). microCLIP (24) algorithm was employed to identify binding events between mRNA peaks and expressed miRNAs. Top expressed miRNAs were retrieved either from (i) the relevant publications, (ii) the analysis of small RNA-Seq (sRNA-Seq) libraries applied to the same cell types/tissues or (iii) databases with miRNA expression profiles across tissues and cell-types (31,32). In datasets with more than one biological replicates, a miRNA binding event had to be present in at least two replicates. Analysis of sRNA-Seq datasets to derive miRNA expression levels was conducted using DIANA-mAP (33).

**CLASH**. Raw Quick CLASH (qCLASH) and CLASH datasets were retrieved and analyzed using Hyb workflow (34) and RNAup from ViennaRNA package (35) for RNA-RNA interaction evaluation. For samples infected with viruses, tailored Hyb databases were built integrating host transcripts, host miRNAs and virally encoded miRNAs. Within each study and condition, a miRNA binding event was annotated as replicated if it exhibited the same transcript coordinates in more than one replicate sample. (q)CLASH interactions were thus annotated regarding the total number of supporting chimeric reads across replicates, the estimated Minimum Free Energy (MFE, kcal/mol) of the binding region and the number of replicates supporting each event. Interactions with a negative MFE, supported by >1 total chimeric reads, were retained.

**RNA-Seq**. Raw RNA-Seq libraries were quality checked and pre-processed using FastQC, minion (36) and cutadapt. Pre-processed reads were aligned to their respective genome for each species using STAR aligner (v2.7.10) (37), followed by execution of RSEM (38) to perform the gene expression quantification step. Differential expression analysis was performed using the programming language R (v4.2.0) and the R package edgeR (v3.42.2) (39). Filtering of low-abundance genes was achieved by employing the filterByExpr function (edgeR) with default arguments. Finally, the weighted trimmed mean of *M*-values (TMM) normalization method and quasi-likelihood *F*-testing were utilized to assess abundance changes between conditions (absolute logFC > 0.58, FDR < 0.05).

**TDMD–sRNA-Seq**. Small RNA-Seq libraries from ZSWIM8 knockout and control samples were quality checked and pre-processed using FastQC, minion and cutadapt. miRNA quantification and differential expression analysis was conducted executing DIANA-mAP at default settings. Publicly available, analyzed, RNA-Seq datasets were retrieved and gene expression measurements of control samples (Transcripts-Per-Million, TPM > 1) were retained for each utilized cell-line. In case of replicates, TPMs were averaged. Candidate TDMD miRNA–gene interactions were formed by integrating (i) significantly up-regulated miRNAs upon ZSWIM8 knockout (i.e. logFC > 0.585 FDR < 0.05) and (ii) protein-coding genes with expression levels above the third quartile of expression in the same condition, which (iii) exhibited direct miRNA-MRE interactions, as evidenced by our in-house analyzed CLIP-Seq datasets.

### Text-mining pipeline

A Natural Language Processing (NLP) pipeline was employed to extract experimentally supported miRNA–gene interactions from PubMed literature. Initially, 4571 sentences were manually labeled as positive (2722 sentences) or irrelevant (1849 sentences). Machine learning was then utilized, specifically employing a BioBERT (40) model for sentiment analysis to classify these sentences. Subsequently, the Stanza model (41) was used to extract dependency trees, and the Term Frequency - Inverse Document Frequency (TF-IDF) statistical method and cosine similarity were applied to capture the relationships between words, particularly those linking keywords and each other. This process facilitated the identification of essential terms in the context of miRNA and gene references, ultimately contributing to the determination of a threshold. This threshold enabled the accurate extraction of interactions from positive sentences. Additionally, the BERN2 tool (42) for Named Entity Normalization (NEN) was integrated into the pipeline. The methodology successfully processed approximately 39 000 papers retrieved through an advanced PubMed query. To assess the quality of the predictions of the NLP model, we manually curated 400 randomly selected abstracts from papers already processed by the method and created a set of miRNA–gene pairs (643 interactions – Supplementary Table 1). Using this set as ground truth, we calculated the True Positive (TP) and False Positive (FP) percentages of the text-mining-derived miRNA–gene interactions. The NLP model exhibited an ∼67% TP rate (i.e. a 33% FP rate). To further refine the abstract-extracted interactions, we utilized a Large Language Model (LLM), specifically gpt-3.5-turbo-0301and used it as an additional filter, applied to the NLP results. A refinement of the initial LLM results involved instructing the LLM to express the miRNA–gene relationships in its own words before translating them into a standardized format for enhanced comprehension. This additional step (LLM filter) yielded an 87.05% TP rate (i.e. a 12.95% FP rate). The total number of predicted interactions corresponds to approximately 19 000 entries originating from ∼15 000 article abstracts.

### Database architecture and implementation

TarBase-v9.0 is a single-page application (SPA) based on React.js (https://github.com/facebook/react) and Tailwind CSS (https://github.com/tailwindlabs/tailwindcss) frameworks. React Router (https://github.com/remix-run/react-router) handles the SPA’s navigation and Recharts (https://github.com/recharts/recharts), Nivo (https://github.com/plouc/nivo) web frameworks are utilized for data visualization. The SPA interacts asynchronously with a Representational State Transfer Application Programming Interface (REST API), which was implemented in the Rust programming language (https://www.rust-lang.org), extending Axum (https://github.com/tokio-rs/axum) framework. The RESTful server communicates with a PostgreSQL relational database (https://github.com/postgres/postgres) via the Diesel Object-Relational Mapper (ORM) (https://github.com/diesel-rs/diesel). The database contains TarBase data in Second and Third Normal Forms (2NF, 3NF) for a manageable number of tables. The queries are optimized by efficient table joining and indices on columns that participate in conditions. Also, the global statistics of the database are cached using Materialized Views.

## Discussion

Meticulous production, curation and indexing of miRNA–gene interactions across various experimental contexts and species is essential for both fundamental and practical RNA research endeavors. TarBase-v9.0 highlights the ongoing commitment to systematically cataloging millions of experimentally supported miRNA targets for over 15 years. The latest version hosts ∼6 million entries, comprising ∼2 million unique miRNA–target pairs, supported by 37 experimental (high- and low-yield) protocols in 172 tissues and cell-types. Importantly, for the first time, it also lists interactions leading to target-directed miRNA degradation (TDMD) events and millions of miRNA-binding locations with cell-type resolution produced by the *de novo* analysis of state-of-the-art NGS methodologies. This content is provided to users through a new interface that introduces numerous new features and enhances its adaptability and user-friendliness.

TarBase v9.0 is the largest collection of experimentally supported miRNA–gene interactions to date. This wealth of information can enforce or even at cases substitute *in silico* predicted interactions.

TarBase may also be accessed here: http://62.217.122.56/.

## Supplementary Material

gkad1071_Supplemental_File

## Data Availability

TarBase is freely available at https://dianalab.e-ce.uth.gr/tarbasev9 and may also be accessed at http://62.217.122.56/. No new data were generated or analysed in support of this research.

## Notes

Present address: Dimitra Karagkouni, Cancer Research Institute | Harvard Medical School, Department of Pathology, Beth Israel Deaconess Medical Center, Boston, MA, USA.

Present address: Dimitra Karagkouni, Harvard Medical School, Boston, MA, USA.
