# Supplementary material for: TarBase-v9.0 extends experimentally supported miRNA–gene interactions to cell-types and virally encoded miRNAs
Source: Nucleic Acids Res. 2023 Nov 20;52(D1):D304–10. doi: 10.1093/nar/gkad1071 (PMC10767993; doi:10.1093/nar/gkad1071)
Supplement: gkad1071_Supplemental_File [file gkad1071_supplemental_file.pdf]

PMID	miRNA name	Gene name
36889771	hsa-miR-525	XIAP
28844709	hsa-miR-491	ABCB1
28806401	hsa-miR-873	GDF15
28806401	hsa-mir-1233-2	GDF15
28844709	hsa-miR-491	SP3
28842285	hsa-miR-935	IFNAR1
28829507	hsa-miR-613	FRS2
28827731	hsa-miR-7641	RPS16
28827731	hsa-miR-7641	TNFSF10
28892647	hsa-miR-96	SOX6
28890396	hsa-miR-525	SH2D3A
28887583	hsa-miR-608	RRM1
28887583	hsa-miR-608	CDA
28882645	hsa-miR-770	JMJD6
28880936	hsa-miR-503	VEGFA
28880489	hsa-miR-4317	ZNF322
28871945	hsa-miR-497	RNF41
28871006	hsa-miR-382	MMP16
28871006	hsa-miR-410	MMP16
28871006	hsa-miR-200b	TIMP2
28860601	hsa-miR-608	CDC42
28859669	hsa-miR-493	FAM168A
28849017	hsa-miR-491	TSPAN1
28844950	hsa-miR-4516	FN1
28844950	hsa-miR-4516	ITGA9
28961507	hsa-miR-622	MYC
28956260	hsa-miR-6165	ABLIM1
28956260	hsa-miR-6165	NECTIN1
28956260	hsa-miR-6165	PKD1
28956260	hsa-miR-6165	PLXNA2
28956260	hsa-miR-6165	PDK1
28943214	hsa-miR-605	EN2
28942143	hsa-miR-503	CDKN2A
28926924	hsa-miR-506	JAG1
28924964	hsa-miR-490	ROBO1
28924385	hsa-miR-449c	MYC
28924102	hsa-miR-483	TGFB1
28912531	hsa-miR-503	PDCD4
28900514	hsa-miR-647	SRF
28893265	hsa-miR-424	LATS1
29028086	hsa-miR-645	DCDC2
29019371	hsa-miR-590	ARHGAP24
29019284	hsa-miR-503	WEE1
28991363	hsa-miR-495	SNAI2
28991363	hsa-miR-495	VMAC
28991363	hsa-miR-495	CDH12
28982084	hsa-miR-484	APAF1
28981108	hsa-miR-451a	YWHAZ
28975989	hsa-miR-577	SPHK2
28972400	hsa-miR-629	TSPYL5

28972040	hsa-miR-708	DNMT3A
28967902	hsa-miR-451a	MYC
28965954	hsa-miR-758	CD36
28965123	hsa-mir-425	CD44
28962647	hsa-miR-524	TP53INP1
28962647	hsa-miR-524	ZEB2
28962647	hsa-miR-524	SMAD4
36004692	hsa-miR-944	NOX4
36002076	hsa-miR-620	IRF2BP2
35996510	hsa-miR-640	SLIT1
35996500	hsa-miR-650	BAX
35993945	hsa-miR-455	CDKN1B
35988100	hsa-miR-625	SCAI
35985176	hsa-miR-494	BIRC5
35980117	hsa-miR-582	ALG3
29054757	hsa-miR-767	CYLD
29048685	hsa-miR-504	TP53INP1
29048659	hsa-miR-520c	CXCL8
29045821	hsa-miR-93	FAT4
29045821	hsa-miR-93	LATS2
29032337	hsa-miR-758	MTOR
29032337	hsa-miR-758	MDM2
36315336	hsa-miR-4735	TNFAIP3
36105681	hsa-miR-559	PARD3
36087740	hsa-miR-621	LEF1
36077039	hsa-miR-99b	AR
36077039	hsa-miR-99b	MTOR
36067529	hsa-miR-6515	DNMT3B
36029209	hsa-miR-637	FGFR1
36027039	hsa-miR-504	IFITM1
36018460	hsa-miR-582	PEG3
36017912	hsa-miR-587	SLIT3
36017148	hsa-miR-96	FHL1
36012303	hsa-let-7d	COL3A1
36012303	hsa-let-7d	SMAD2
36007337	hsa-miR-767	ASF1B
36004693	hsa-miR-576	NEGR1
36210706	hsa-let-7g	HMGB2
36206112	hsa-miR-497	SLC1A5
36202224	hsa-miR-424	VWF
36200682	hsa-miR-421	SIRT3
36184551	hsa-miR-6870	TOLLIP
36181561	hsa-miR-634	MAP2K4
36164322	hsa-miR-452	TIMP3
36156852	hsa-miR-506	AKT2
36156291	hsa-miR-599	MYD88
36153332	hsa-miR-760	HM13
36128051	hsa-miR-524	MEF2C
36125054	hsa-miR-944	MAD2L1
36115716	hsa-miR-373	CD44
36115716	hsa-miR-520a	CD44

36115716	hsa-miR-520b	CD44
36215729	hsa-miR-6838	CBX4
36219319	hsa-miR-429	MYC
36219319	hsa-miR-429	BCL2
36219319	hsa-miR-429	EGFR
36224238	hsa-miR-936	ANXA2
36239568	hsa-miR-936	GPR78
36248191	hsa-miR-652	TP53
36250452	hsa-miR-574	IL6
36252912	hsa-miR-451a	DCBLD2
36252912	hsa-miR-451a	AKT1
36260266	hsa-miR-633	MAPK1
36266695	hsa-miR-874	SOX12
36289359	hsa-miR-4653	HIPK2
36274974	hsa-miR-7847	PRKCG
36274500	hsa-miR-501	TM4SF1
36274378	hsa-miR-433	E2F3
36272152	hsa-miR-650	KLF12
36272150	hsa-miR-873	KLF5
36268034	hsa-miR-585	CREB1
36268034	hsa-miR-585	MAPK1
36268034	hsa-miR-585	MITF
36326978	hsa-miR-622	HOXA9
36323841	hsa-miR-874	PMVK
36323841	hsa-miR-874	SREBF2
36309967	hsa-miR-548k	PTEN
36305147	hsa-miR-424	CCNE1
36301034	hsa-miR-762	GIPC3
36299824	hsa-miR-452	RGS2
36481473	hsa-miR-936	CUL4B
36473369	hsa-miR-96	LDB2
36456979	hsa-miR-576	CAND1
36456979	hsa-miR-132	CAND1
36456979	hsa-miR-132	ZDHHC23
36456979	hsa-miR-576	ZDHHC23
36456979	hsa-miR-132	AHR
36456979	hsa-miR-576	AHR
36456979	hsa-miR-132	MTMR4
36456979	hsa-miR-576	MTMR4
36444911	hsa-miR-708	RAP2B
36441004	hsa-miR-93	PAFAH1B1
36423859	hsa-miR-4306	PAK6
36401721	hsa-miR-506	GSTP1
36385135	hsa-miR-574	PEG10
36369960	hsa-miR-493	CARTPT
36598315	hsa-miR-568	RNF38
36578050	hsa-miR-4443	PTPRJ
36555553	hsa-miR-503	WNT3A
36538280	hsa-miR-576	ZBTB4
36535407	hsa-miR-495	ABCB1
36527891	hsa-miR-483	IGF1

36512648	hsa-miR-655	CXCR4
36504050	hsa-miR-607	TRPC5
36482739	hsa-miR-455	H2AZ1
36727232	hsa-miR-424	KIF23
36705418	hsa-miR-525	BAX
36629518	hsa-miR-6742	FGF8
36624091	hsa-miR-488	PLAG1
36623770	hsa-miR-98	HBEGF
36871032	hsa-miR-424	SPAG7
36871032	hsa-miR-15b	SPAG7
36871032	hsa-miR-195	SPAG7
36871032	hsa-miR-497	SPAG7
36841132	hsa-miR-519c	MECP2
36793891	hsa-miR-491	SLIT3
36750722	hsa-miR-582	PDHB
36750722	hsa-miR-582	DKK1
36750173	hsa-miR-874	VDAC1
36737832	hsa-miR-455	PDZK1IP1
36737507	hsa-let-7c	CDCA8
36737402	hsa-miR-874	ENAH
36734858	hsa-miR-873	ROCK1
31646577	hsa-miR-409	NUP43
30873717	hsa-miR-944	GATA6
25776495	hsa-miR-411	ITCH
26455324	hsa-let-7c	IL6
26455324	hsa-let-7c	IL6R
26455324	hsa-let-7c	IGF1R
26455324	hsa-miR-99a	IL6
26455324	hsa-miR-99a	IL6R
26455324	hsa-miR-99a	IGF1R
26455324	hsa-miR-125b	IL6
26455324	hsa-miR-125b	IL6R
26455324	hsa-miR-125b	IGF1R
31182803	hsa-miR-503	ELK3
31182803	miR-4269	ELK3
31182803	hsa-miR-30e	ELK3
33121976	hsa-miR-642a	LGMN
36929708	hsa-miR-513b	JPT1
36810285	hsa-miR-17	MFN2
36562476	hsa-miR-761	DACT2
36228497	hsa-miR-135a-1	BAG3
36228497	hsa-miR-135a-2	BAG3
36206097	hsa-miR-596	NKAP
27160903	hsa-miR-551b	STAT3
25980492	hsa-miR-659	CNOT1
36154592	hsa-miR-122	ABCC1
36045117	hsa-miR-365a	RAC1
36045117	hsa-miR-145	SOX2
35993943	hsa-miR-488	ROCK1
35673915	hsa-miR-744	CCND1
35659199	hsa-miR-199b	MYRF

35576835	hsa-miR-6884	DVL3
35545336	hsa-miR-133b	SOX4
35474604	hsa-miR-1261	PIK3CA
35471148	hsa-miR-139	CBX3
35358000	hsa-miR-584	YAP1
35356749	hsa-miR-370	IL6
35325509	hsa-miR-1260b	CASP8
35276698	hsa-miR-1272	SCARB1
35274813	hsa-miR-582	YAP1
35263216	hsa-miR-300	ASF1B
35256570	hsa-miR-5194	SOX12
35237695	hsa-miR-582	TUG1
35237695	hsa-miR-582	SIGLEC15
33726686	hsa-miR-503	PDCD4
30923258	hsa-miR-506	ZEB2
29518546	hsa-miR-184	PCARE
31438886	hsa-miR-6778	YWHAE
33144585	hsa-miR-671	NFIA
23915286	hsa-miR-433	TYMS
33734890	hsa-let-7i	HOXA9
33734890	hsa-miR-495	PIK3R1
33734890	hsa-miR-495	CDK6
31013711	hsa-miR-590	FOXO3
31013711	hsa-miR-590	CCNG2
23211491	hsa-miR-98	ACVR1B
23211491	hsa-miR-98	MMP11
25234715	hsa-miR-99a	MMP7
25234715	hsa-miR-99a	MMP13
28393215	hsa-miR-539	FSCN1
30229828	hsa-miR-451a	ATF2
25405810	hsa-miR-449a	HDAC1
33822442	hsa-miR-646	STAT3
29343703	hsa-miR-6852	FOXM1
33556191	hsa-miR-152	TGFA
26567912	hsa-miR-203a	PLD2
26567912	hsa-miR-887	PLD2
26567912	hsa-miR-3619	PLD2
26567912	hsa-miR-182	PLD1
30250022	hsa-miR-150	VEGFA
23884313	hsa-miR-203a	TYR
23884313	hsa-miR-203a	KIF5B
23884313	hsa-miR-203a	CREB1
23884313	hsa-miR-203a	MLANA
31124343	hsa-miR-27b	IL1B
31124343	hsa-miR-27b	TNF
28433657	hsa-miR-938	PHLPP2
28433657	hsa-miR-938	CCND1
28433657	hsa-miR-938	MYC
25012295	hsa-miR-26a-1	MAP3K2
24141785	hsa-miR-9-1	CXCR4
29575368	hsa-miR-708	ZEB1

29575368	hsa-miR-708	CDH2
29575368	hsa-miR-708	VIM
32130794	hsa-miR-340	SOX2
35188404	hsa-miR-298	TCF4
31831098	hsa-miR-15a	HMGA1
31831098	hsa-miR-15a	HMGA2
31831098	hsa-miR-15b	HMGA1
31831098	hsa-miR-15b	HMGA2
31831098	hsa-miR-16-1	HMGA1
31831098	hsa-miR-16-1	HMGA2
30847933	hsa-miR-3184	FOXP4
30847933	hsa-miR-181c	PPARA
31181445	hsa-miR-1197	HOXC11
22751122	hsa-miR-143	KRAS
22751122	hsa-miR-145	KRAS
32756011	hsa-miR-195	VEGFA
26902120	hsa-miR-130b	NKD2
24280681	hsa-miR-204	SNAI2
24280681	hsa-miR-204	CDH1
24280681	hsa-miR-204	VIM
26593208	hsa-miR-9-1	FOXO1
28902349	hsa-miR-144	EZH2
31630447	hsa-miR-146a	IRAK1
31630447	hsa-miR-146b	IRAK1
31630447	hsa-miR-146a	CARD10
31630447	hsa-miR-146b	CARD10
25109742	hsa-miR-101-1	PTGS2
30089483	hsa-miR-363	SPAG5
32964027	hsa-miR-587	RPSA
35150890	hsa-miR-485	SLC23A2
35113040	hsa-miR-29a	STAT3
35106926	hsa-miR-142	MAP4K3
28290615	hsa-miR-214	RASSF5
35100076	hsa-miR-149	INHBA
34957298	hsa-miR-149	FOXC1
34828307	hsa-miR-1206	ESRP1
26079799	hsa-miR-199a-1	CD44
26079799	hsa-miR-199a-2	CD44
32783743	hsa-miR-23a	ECM1
23631646	hsa-miR-376c	TGFA
31837329	hsa-miR-193a	TSPAN3
31836508	hsa-miR-130a	SKIL
34780054	hsa-miR-29c	CYP1A2
34780054	hsa-miR-29c	AHR
34780054	hsa-miR-653	CYP1A2
34780054	hsa-miR-653	AHR
34672248	hsa-miR-15a	PDCD4
33179372	hsa-miR-485	BMP7
25370363	hsa-miR-144	AKT3
32583079	hsa-miR-1277	COL5A1
30015869	hsa-miR-577	WNT2B

29876362	hsa-miR-221	PTEN
27471108	hsa-miR-423	ING4
32443852	hsa-miR-1253	WASF2
28982593	hsa-miR-382	DLC1
26078353	hsa-miR-144	HSF2
25119599	hsa-miR-152	CD151
29955039	hsa-miR-29c	ASB2
30057315	hsa-miR-654	IGF2BP3
25613642	hsa-miR-376a-1	HDAC9
24285464	hsa-miR-141	TM4SF1
31951319	hsa-miR-646	CCND1
23707559	hsa-miR-138-1	EZH2
23707559	hsa-miR-138-1	CDK6
23707559	hsa-miR-138-1	E2F2
23707559	hsa-miR-138-1	E2F3
29473240	hsa-miR-590	PPM1F
25725194	hsa-miR-491	KDM4B
30205366	hsa-miR-21	PDCD4
26163618	hsa-miR-99a	MTOR
27641336	hsa-miR-182	PDK4
29862445	hsa-miR-96	MTOR
29862445	hsa-miR-557	RPS6KB1
29862445	hsa-miR-3182	MTOR
29862445	hsa-miR-3182	RPS6KB1
32675387	hsa-miR-153-1	MCL1
32675387	hsa-miR-153-2	MCL1
26065921	hsa-miR-27b	ENPP1
23869586	hsa-miR-200c	ZEB1
30503385	hsa-miR-125b-1	BAK1
25934693	hsa-miR-19	TGM2
29393891	hsa-miR-34a	MDM4
29393891	hsa-miR-34a	CASP9
29393891	hsa-miR-34a	CASP3
28423620	hsa-miR-940	SRC
21219875	hsa-miR-328	ABCG2
21219875	hsa-miR-519c	ABCG2
23034448	hsa-miR-27a	TAB3
23034448	hsa-miR-27a	MAP2K4
23034448	hsa-miR-27a	MAP2K7
23034448	hsa-miR-27a	MAPK14
23034448	hsa-miR-27a	MAPK1
30558790	hsa-miR-181a-2	PPARA
30558790	hsa-miR-181a-1	PPARA
19701194	hsa-miR-26a-1	IL6
32073729	hsa-miR-136	NOTCH3
34667156	hsa-miR-4523	PGK1
34664776	hsa-miR-625	WEE1
31823518	hsa-miR-338	NFATC1
30903795	hsa-miR-744	TGFB1
23157748	hsa-miR-383	PRDX3
24891298	hsa-miR-203a	NANOG

24891298	hsa-miR-203a	POU5F1
28627030	hsa-miR-375	SLC7A11
23239100	hsa-miR-21	SPRY2
33982790	hsa-miR-454	FOXJ2
31509760	hsa-miR-1245b	GKN1
31509760	hsa-miR-544a	GKN2
32587378	hsa-miR-92a-2	AR
29618288	hsa-miR-212	FOXM1
29843929	hsa-miR-4497	GBX2
27495872	hsa-miR-187	CYP1B1
32626991	hsa-miR-34a	NOTCH1
31648164	hsa-miR-383	STAT3
31599417	hsa-miR-29c	CDK6
29637273	hsa-miR-21	PELI1
29637273	hsa-miR-21	SPRY2
26787707	hsa-miR-140	PPP1R13L
26787707	hsa-miR-140	MMP2
26787707	hsa-miR-140	MMP9
24040137	hsa-miR-203a	PRKCA
27448976	hsa-miR-320a	USP14
27448976	hsa-miR-320a	VIM
32980991	hsa-miR-142	MELK
24638856	hsa-miR-2278	IL22RA2
24638856	hsa-miR-411	IL22RA2
27188727	hsa-miR-24-1	XIAP
27188727	hsa-miR-24-2	XIAP
23454155	hsa-miR-218-1	CAV2
23454155	hsa-miR-218-2	CAV2
26657485	hsa-miR-142	WASL
26657485	hsa-miR-142	ITGAV
26657485	hsa-miR-142	RAC1
26657485	hsa-miR-142	CFL2
26657485	hsa-miR-142	ROCK2
26657485	hsa-miR-142	IL6ST
26657485	hsa-miR-142	KLF4
26657485	hsa-miR-142	PGRMC2
26657485	hsa-miR-142	ADCY9
25473903	hsa-miR-218-1	BIRC5
25473903	hsa-miR-218-2	BIRC5
26788506	hsa-miR-221	MBD2
34661273	hsa-miR-370	EZH1
34552061	hsa-miR-9-1	MTDH
34552061	hsa-miR-9-2	MTDH
24690174	hsa-miR-21	LRRFIP1
30605519	hsa-miR-484	PAX5
30605519	hsa-miR-210	PAX5
33201900	hsa-miR-424	E2F7
19767772	hsa-miR-126	CRK
25823465	hsa-miR-1246	DYRK1A
30208330	hsa-miR-146a	CXCR4
30208330	hsa-miR-146a	SMAD4

30225271	hsa-miR-125a	TXNRD1
26023735	hsa-miR-15b	RAB1A
30429198	hsa-miR-584	ROCK1
25365510	hsa-miR-21	PTEN
32106859	hsa-miR-522	ALOX15
32552939	hsa-miR-425	MEIS1
28760781	hsa-miR-432	KEAP1
31668113	hsa-miR-491	HMGA2
31215650	hsa-miR-18b	DOCK4
28075453	hsa-miR-375	HOXB3
27857177	hsa-miR-96	PTPN9
33221743	hsa-miR-34a	HNF4G
33221743	hsa-miR-34a	NOTCH1
32155139	hsa-miR-142	CDK6
29236292	hsa-miR-4301	DRD2
30938104	hsa-miR-204	HMGA2
33882454	hsa-miR-3182	KLF4
25592039	hsa-miR-29b-1	IGF1
25592039	hsa-miR-29b-2	IGF1
33705812	hsa-miR-21	VEGF
33705812	hsa-miR-21	PTEN
33705812	hsa-miR-21	TGFBR2
33705812	hsa-miR-301a	VEGF
33705812	hsa-miR-301a	PTEN
33705812	hsa-miR-301a	TGFBR2
32266659	hsa-miR-186	CDK6
32266659	hsa-miR-186	BCL2
32266659	hsa-miR-186	CCND1
30871575	hsa-miR-338	IL6
31884339	hsa-miR-32	DNAJB9
22962603	hsa-miR-214	EZH2
22962603	hsa-miR-214	CTNNB1
28961027	hsa-let-7i	BAG1
26398931	hsa-miR-155	FOXO3
20933503	hsa-miR-218-1	IKBKB
20933503	hsa-miR-218-1	MMP9
20933503	hsa-miR-218-2	IKBKB
20933503	hsa-miR-218-2	MMP9
25951106	hsa-miR-145	SOX2
27888625	hsa-miR-24-1	TRIM11
27888625	hsa-miR-24-2	TRIM11
28381166	hsa-miR-181d	NKAIN2
23403580	hsa-miR-483	IGF1
27405111	hsa-miR-638	SMC1A
34532813	hsa-miR-432	BCL2
34429120	hsa-miR-499a	UBE2V2
34382916	hsa-miR-527	YWHAZ
34365722	hsa-miR-616	DUSP2
34357507	hsa-miR-582	TNKS2
34318539	hsa-miR-106b	TXNIP
34279157	hsa-miR-143	MARCKS

34162560	hsa-mir-93	PTEN
31564215	hsa-miR-122	ALDOA
32132656	hsa-miR-27a	AMPK
26160756	hsa-miR-1275	IGF2BP1
26160756	hsa-miR-1275	IGF2BP2
26160756	hsa-miR-1275	IGF2BP3
19638978	hsa-miR-143	DNMT3A
34016788	hsa-miR-454	TNF
17600087	hsa-let-7a-1	HMGA2
17600087	hsa-let-7a-2	HMGA2
17600087	hsa-let-7a-3	HMGA2
33892654	hsa-miR-454	UBB
26758433	hsa-miR-143	FAM83F
25754817	hsa-miR-5100	RAB6A
25754817	hsa-miR-5100	CCND1
25754817	hsa-miR-5100	CDK2
31252267	hsa-miR-34a	HMGB1
19437538	hsa-miR-125b-1	VDR
19437538	hsa-miR-125b-2	VDR
24440705	hsa-miR-490	CCND1
28627703	hsa-miR-495	HMGN5
28627703	hsa-miR-495	CCNB1
28627703	hsa-miR-495	BCL2
28627703	hsa-miR-495	MMP9
25088422	hsa-miR-638	PTEN
25088422	hsa-miR-638	TP53
25088422	hsa-miR-518c	PTEN
25088422	hsa-miR-518c	TP53
34147851	hsa-miR-934	PTEN
32950571	hsa-miR-98	IGF1
15538371	hsa-miR-375	MTPN
15806104	hsa-miR-375	MTPN
15806104	hsa-let-7b	MTPN
15944709	hsa-miR-17	E2F1
15944709	hsa-miR-20a	E2F1
16549775	hsa-miR-130a	MAFB
16549775	hsa-miR-10a	HOXA1
17322061	hsa-miR-376a-1	PRPS1
17404574	hsa-miR-29b-1	MCL1
17404574	hsa-miR-29b-2	MCL1
18728182	hsa-miR-19b-2	SOCS1
17616664	hsa-miR-122a	CCNG1
17627278	hsa-miR-222	CDKN1B
17627278	hsa-miR-221	CDKN1B
17668390	hsa-miR-155	AGTR1
17681183	hsa-miR-21	PTEN
17855557	hsa-miR-130a	TAC1
17855557	hsa-miR-206	TAC1
17914108	hsa-miR-222	CDKN1B
17914108	hsa-miR-221	CDKN1B
18197755	hsa-mir-26a-1	SMAD1

18212054	hsa-miR-106b	CDKN1A
18328430	hsa-miR-106b	E2F1
18328430	hsa-miR-93	E2F1
18376396	hsa-miR-205	ZEB1
18376396	hsa-miR-200a	ZEB1
18376396	hsa-miR-200b	ZEB1
18376396	hsa-miR-200c	ZEB1
18376396	hsa-miR-205	ZEB2
18376396	hsa-miR-200a	ZEB2
18376396	hsa-miR-200b	ZEB2
18376396	hsa-miR-200c	ZEB2
18381414	hsa-miR-137	E2F6
18381414	hsa-miR-193a	CDK6
18663355	hsa-miR-196a-1	ANXA1
18663355	hsa-miR-196a-2	ANXA1
18728182	hsa-miR-19a	SOCS1
18728182	hsa-miR-19b-1	SOCS1
18728182	hsa-miR-106b	TAF6L
18728182	hsa-miR-181a-1	TAF6L
18728182	hsa-miR-181a-2	TAF6L
18728182	hsa-miR-32	TAF6L
18728182	hsa-miR-181b-1	TAF6L
18728182	hsa-miR-181b-2	TAF6L
19703567	hsa-miR-124-1	BDNF
19703567	hsa-let-7d	DRD3
19703567	hsa-miR-124-2	BDNF
21220473	hsa-miR-125a	ERBB2
21937511	hsa-miR-122	SLC7A1
21937511	hsa-miR-122	ADAM17
21937511	hsa-miR-122	BCL2L2
21937511	hsa-miR-122	PRKRA
22005523	hsa-miR-130b	CSF1
22102825	hsa-miR-34a	ARAF
22102825	hsa-miR-34a	PIK3R2
22102825	hsa-miR-34a	SMAD4
22102825	hsa-miR-34a	PLK1
22102825	hsa-miR-34a	MCM5
22102825	hsa-miR-34a	MCM2
22102825	hsa-miR-34a	CCND3
22102825	hsa-miR-34a	CCNG2
22158624	hsa-miR-21	DOCK4
22158624	hsa-miR-21	DOCK5
22158624	hsa-miR-21	DOCK7
22294051	hsa-miR-221	GJA1
22294051	hsa-miR-222	GJA1
22323518	hsa-miR-130a	MAFB
22454525	hsa-miR-378a	NODAL
22649212	hsa-miR-23b	VHL
22952885	hsa-miR-19a	IMPDH1
22952885	hsa-miR-19a	NPEPL1
23451058	hsa-miR-19a	SUZ12

23451058	hsa-miR-19a	RAB13
23451058	hsa-miR-19a	MSMO1
23451058	hsa-miR-19a	PSAP
23451058	hsa-miR-19a	ABCA1
23451058	hsa-miR-27a	ABCA1
23451058	hsa-miR-27a	PDS5B
23451058	hsa-miR-133b	CDK13
23451058	hsa-miR-133b	PTPRK
23451058	hsa-miR-133b	RB1CC1
23451058	hsa-miR-133b	CPNE3
23471579	hsa-miR-216a	PTEN
23471579	hsa-miR-216a	SMAD7
23471579	hsa-miR-217	PTEN
23471579	hsa-miR-217	SMAD7
23503464	hsa-miR-99a	IGF1R
23503464	hsa-let-7c	IGF1R
23503464	hsa-miR-125b-2	IGF1R
23544130	hsa-miR-497	CCNE1
23544130	hsa-miR-497	CDC25A
23544130	hsa-miR-497	CCND3
23544130	hsa-miR-497	CDK4
23544130	hsa-miR-497	BTRC
23544130	hsa-miR-195	CCNE1
23544130	hsa-miR-195	CDC25A
23544130	hsa-miR-195	CCND3
23544130	hsa-miR-195	CDK4
23544130	hsa-miR-195	BTRC
23552692	hsa-miR-375	CIP2A
23789915	hsa-miR-494	PROS1
23816858	hsa-miR-124	CD151
23922722	hsa-miR-376c	GRB2
23955600	hsa-miR-200a	NOTCH1
23955600	hsa-miR-200b	NOTCH1
23955600	hsa-miR-200c	NOTCH1
23955600	hsa-miR-34b	NOTCH1
23955600	hsa-miR-34c	NOTCH1
23969726	hsa-miR-222	KIT
23969726	hsa-miR-17	ETV1
23969726	hsa-miR-20a	ETV1
24033605	hsa-miR-143	HK2
24033605	hsa-miR-145	HK2
24040069	hsa-miR-4437	SNCG
24040069	hsa-miR-4674	SNCG
24040069	hsa-miR-885	SNCG
24040069	Hsa-miR-138-1	SNCG
24040069	hsa-miR-497	SNCG
24147004	hsa-miR-525	ARRB1
24147004	hsa-miR-525	TXN1
24147004	hsa-miR-525	HSPA9
24312276	hsa-miR-126	IRS1
24337069	hsa-miR-200c	SEC23A

24337069	hsa-miR-200b	SEC23A
24337069	hsa-miR-375	SEC23A
24337069	hsa-miR-375	PHLPP1
24337069	hsa-miR-15a	PHLPP2
24577056	hsa-miR-214	CD276
24577056	hsa-miR-363	CD276
24577056	hsa-miR-326	CD276
24577056	hsa-miR-940	CD276
24577056	hsa-miR-29c	CD276
24577056	hsa-miR-665	CD276
24577056	hsa-miR-34b	CD276
24577056	hsa-miR-708	CD276
24577056	hsa-miR-601	CD276
24577056	hsa-miR-124-1	CD276
24577056	hsa-miR-380	CD276
24577056	hsa-miR-885	CD276
24577056	hsa-miR-593	CD276
24769899	hsa-miR-615	IGF2
24769899	hsa-miR-615	JUNB
24777293	hsa-miR-145	ABCG2
24793973	hsa-miR-31	ARID1A
24884732	hsa-miR-182	SATB2
24914051	hsa-miR-382	PTEN
24920276	hsa-miR-17	ADAR
24938624	hsa-miR-21	CCL20
24970812	hsa-miR-34a	YY1
24970812	hsa-miR-34b	YY1
24970812	hsa-miR-34c	YY1
24997798	hsa-miR-200c	USP25
25041637	hsa-miR-106b	PTEN
25138213	hsa-miR-326	HDAC3
25634213	hsa-miR-29c	CTNND1
